# How Does Faith Impact End of Life Care Preferences Among Older Chinese in the United States: A Qualitative Pilot Study

**DOI:** 10.1093/geroni/igaf122.3603

**Published:** 2025-12-31

**Authors:** Xuehan Zhang, Amanda Woodward

**Affiliations:** Michigan State University, East Lansing, Michigan, United States; Michigan State University, East Lansing, Michigan, United States

## Abstract

Previous studies have found that religious participation is closely related to older people’s overall well-being and life satisfaction. However, the specific impact of religion on older individuals’ end-of-life care preferences and death attitudes from racial minority groups remains underexplored. This qualitative pilot study aims to better understand the impact of religion (e.g., Christianity) on beliefs and preferences related to end-of-life care services among older Chinese adults in the United States, particularly those who are not immediately facing death or the need to make these care decisions. 13 semi-structured interviews were conducted in Ann Arbor, Michigan during the summer of 2025. All interviews were in Chinese and then translated into English for coding and analysis. Each interview was coded in Dedoose. Three main findings were identified: 1) Participants in the present study indicated that having no fear of death, not only because of their religious beliefs, but also because of their great confidence in the medical care services in the United States. 2) Participants in the present study were open to talking about euthanasia as a potential option for treatment in the end. 3) Most people who were interviewed in the present study lacked information about end-of-life care options but were willing to go to the “hospital” to receive care in the last stage of life. These findings highlight a critical need for culturally and religiously sensitive end-of-life care services and education among the older Chinese population in the United States.

